# Aetiology of Bacteraemia as a Risk Factor for Septic Shock at the Onset of Febrile Neutropaenia in Adult Cancer Patients

**DOI:** 10.1155/2014/561020

**Published:** 2014-03-20

**Authors:** Regis Goulart Rosa, Luciano Zubaran Goldani

**Affiliations:** Infectious Diseases Unit, Infectious Diseases Division of Hospital de Clínicas de Porto Alegre, Universidade Federal do Rio Grande do Sul, Ramiro Barcelos 2350, Room 700, Porto Alegre 90640-000, RS, Brazil

## Abstract

Septic shock (SS) at the onset of febrile neutropaenia (FN) is an emergency situation that is associated with high morbidity and mortality. The impact of the specific aetiology of bloodstream infections (BSIs) in the development of SS at the time of FN is not well established. The aim of this study was to evaluate the association between the aetiology of BSIs and SS at the time of FN in hospitalised adult cancer patients. This prospective cohort study was performed at a single tertiary hospital from October 2009 to August 2011. All adult cancer patients admitted consecutively to the haematology ward with FN were evaluated. A stepwise logistic regression was conducted to verify the association between the microbiological characteristics of BSIs and SS at the onset of FN. In total, 307 cases of FN in adult cancer patients were evaluated. There were 115 cases with documented BSI. A multivariate analysis showed that polymicrobial bacteraemia (*P* = 0.01) was associated with SS. The specific blood isolates independently associated with SS were viridans streptococci (*P* = 0.02) and *Escherichia coli* (*P* = 0.01). Neutropaenic cancer patients with polymicrobial bacteraemia or BSI by viridans streptococci or *Escherichia coli* are at increased risk for SS at the time of FN.

## 1. Introduction

Despite improvements in treating febrile neutropaenia (FN) and sepsis over the past decade, septic shock (SS) continues to be associated with substantial morbidity and mortality among cancer patients undergoing intensive cytotoxic chemotherapy [1]. The unpredictable clinical course of infections in neutropaenic patients because of the lack of an adequate inflammatory response makes managing FN a significant challenge because clinically stable patients may suddenly progress to severe sepsis or SS [2].

SS is a result of the host response to the pathogen and is dependent on the virulence of the microorganism and the infection site [3]. The known risk factors for SS in immunocompetent patients include advanced age, low functional status, and the presence of cancer, clinical comorbidities, nosocomial infections, and infection that does not originate in the urinary tract [4–6]. Infection with certain bacteria, such as* Staphylococcus aureus* and* Pseudomonas aeruginosa*, is also associated with an increased risk for SS, as the expression of certain proteins or molecules (virulence factors) contributes to pathogen replication and dissemination by subverting or eluding the host's defences [7]. Unfortunately, data regarding the influence of microbiological factors on the development of SS in cancer patients with FN is scarce. Therefore, we conducted a study with the aim of evaluating the association between microbiological aspects of bloodstream infections (BSIs) and SS development at the onset of FN in hospitalised adult cancer patients.

## 2. Methods

### 2.1. Study Design and Participants

A prospective cohort study was conducted at a single referral centre for adult bone marrow transplantation in Southern Brazil from October 2009 to August 2011. This study followed all consecutive haemodynamically stabile cancer patients older than 18 years of age who were admitted to the haematology ward of the Hospital de Clínicas de Porto Alegre (Porto Alegre, Brazil) with neutropaenia (i.e., an absolute neutrophil count (ANC) < 500 cells/mm^3^ or <1000 cells/mm^3^ with an expectation of a decrease to <500 cells/mm^3^ during the ensuing 48 h). The subjects who developed fever (i.e., a single axillary temperature measurement ≥38.5°C or sustained temperature ≥38.0°C over a 1 h period) during the course of neutropaenia were entered into the study. Outpatients, patients who had neutropaenia caused by a specific aetiology other than an adverse reaction to chemotherapy, and patients who had episodes of FN without documented bacteraemia were excluded. Subjects were allowed to reenter the study after an initial episode of FN if they remained free of signs or symptoms of infection for at least 7 days after completing the treatment for the first episode and if all causative organisms, if any, were eradicated.

### 2.2. Definitions

Microbiological studies, which included 2 separate blood samples that were obtained from 2 different anatomical sites for culture, were performed at the onset of fever, according to standard practice. In the absence of an indwelling central venous catheter, 2 blood samples were obtained from 2 distinct peripheral veins. When an indwelling central venous catheter was present, 1 blood sample was obtained through this catheter, and a second sample was obtained from a peripheral vein. The susceptibilities of the isolated pathogens to antibiotics were evaluated according to the recommendations of the Clinical and Laboratory Standards Institute [8]. Bacteraemia caused by coagulase-negative* Staphylococcus* spp. was diagnosed after 2 positive results from 2 independent cultures. Bacteraemia indicated by 1 positive culture was considered to be diagnostic for the other microorganisms. Polymicrobial BSI was characterised as a bacteraemic episode due to at least two different pathogens isolated from the same blood sample. Multidrug-resistant (MDR) bacteraemia was defined as a BSI with methicillin-resistant staphylococci or vancomycin-resistant enterococci for Gram-positive bacteria or as resistance to ≥3 classes of antimicrobial agents for Gram-negative bacteria. Clinical comorbidity was determined by the presence of heart failure, diabetes mellitus, chronic pulmonary disease, chronic liver disease, or chronic renal failure. Profound neutropaenia was characterised by an ANC < 100 cells/mm^3^. The patients were divided into 2 groups based on their chemotherapy regimens: a high-dose chemotherapy group that included patients who underwent haematopoietic stem cell transplantation or induction chemotherapy and a standard-dose chemotherapy group that included patients who underwent consolidation or maintenance chemotherapy. Nosocomial-acquired FN was defined as the onset of FN after 48 hours of hospitalisation. The oral mucositis grade was classified according to the World Health Organisation's oral toxicity scale [9].

### 2.3. Outcomes and Followup

The primary outcome measure of the present study was SS at the onset of fever in neutropaenic patients. SS was defined as persistent haemodynamic instability (systolic arterial pressure <90 mmHg or a reduction in systolic blood pressure >40 mmHg from baseline) despite adequate fluid resuscitation (30 mL per Kg of crystalloid) with at least 2 systemic inflammatory response syndrome criteria [10]. The secondary outcome was mortality by day 28. Researchers who were not associated with the assistant physician's team conducted the patient followups through interviews and medical record reviews using a standardised data collection instrument. The followup was maintained for 28 days after the onset of fever in the neutropaenic patients. For the subjects who were discharged before 28 days, follow-up telephone calls were made on the 28th day after the onset of FN to determine whether they remained alive; if a patient was deceased at the time of the phone call, the survival time was calculated based on the date of death reported by the family.

### 2.4. Statistical Analysis

Stepwise logistic regression analysis was performed to determine whether the microbiological characteristics of BSIs were risk factors for SS at the time of FN. All clinical and microbiological variables that had a *P* value <0.10 in the univariate analysis were included. In the multivariate model, independent variables were eliminated from the highest to the lowest *P* value but remained in the model if the *P* value was <0.05. Odds ratios (OR) were estimated with 95% confidence intervals (95% CI). Kaplan-Meier curves were utilised to evaluate the time-dependent occurrence of death; the log-rank test was applied for between-group comparisons. The statistical analysis was performed using STATA version 12 (Stata Corp LP, USA).

### 2.5. Ethics Statement

Written informed consent was obtained from all study participants. The institutional review board of the Hospital de Clínicas de Porto Alegre approved the study.

## 3. Results

In total, 307 episodes of FN (in 169 patients) were evaluated; a total of 115 BSIs were documented (37.4% of all episodes). Antibiotic prophylaxis was not administered to any patient. The incidence of SS was 14.7% (17 episodes).

The characteristics of the study population and the specific pathogens responsible for all BSIs in the present cohort are shown in Table 1. Subjects with haematological malignancies comprised 83.5% of the study population; haematopoietic stem cell transplantation was performed in 21.7% of the cases. Forty-eight percent of the study sample had some degree of chemotherapy-induced mucositis. The proportion of nosocomial episodes of FN was 88.7%. In descending order, the predominant blood isolates were* Escherichia coli*, coagulase-negative staphylococci,* Klebsiella pneumoniae*,* Pseudomonas aeruginosa*, viridans streptococci, and* Enterococcus* spp. Among all BSIs evaluated, 38 cases were due to MDR bacteria (Table 2): 4 cases in the SS group (23.5%) and 34 cases in the non-SS shock group (34.6%). Methicillin resistance and the production of extended-spectrum beta-lactamase were the most frequent types of antimicrobial resistance, occurring in 96.2% of BSIs involving Gram-positive MDR bacteria and 83.3% of BSIs involving Gram-negative MDR bacteria, respectively.

A univariate analysis revealed that polymicrobial BSI (*P* = 0.01) and bacteraemia by* Escherichia coli* (*P* = 0.04) were associated with the main outcome (Table 3). Multidrug-resistant (MDR) bacteraemia was not associated with SS at the onset of FN with either the Gram-positive MDR or Gram-negative MDR bacteria.

After multiple logistic regression analyses were performed (Table 4, model 1), the only variable that constituted an independent risk factor for SS at the time of FN was polymicrobial BSI (OR, 5.41, 95% CI, 1.48–19.79). A second logistic regression model was used to assess the effect of specific pathogens on the development of SS without the inclusion of other microbiological variables (Table 4, model 2). This model was conducted to avoid the dilution of the effect of specific pathogens by other microbiological factors in the multivariate analysis. The specific blood isolates that were independently associated with the main outcome were viridans streptococci (OR, 7.58, 95% CI, 1.34–42.80) and* Escherichia coli* (OR, 4.30, 95% CI, 1.34–14.48). The percentage of the polymicrobial samples that included* E. coli* and viridans streptococci was 58.3% (7 cases) and 25% (3 cases), respectively.

**Figure 1:**
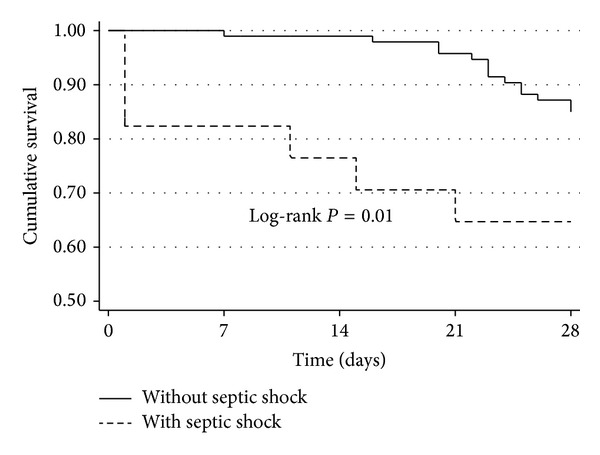
Survival curves according to the presence of septic shock at the time of febrile neutropenia in hospitalised adult cancer patients.

As expected, the 28-day mortality rate of the patients who presented with SS at the time of FN was greater than that of the patients who did not present with SS (35.2% versus 14.2%, log-rank *P* = 0.01) (Figure 1).

## 4. Discussion

The present prospective cohort study demonstrated that cancer patients with polymicrobial bacteraemia were more likely to develop SS at the onset of FN. In particular, BSIs involving* E. coli* and viridans streptococci were independently associated with SS at the time of FN. The 28-day survival rate of the patients with SS at the time of FN was significantly lower than that of the patients who did not present with SS.

Previous observational studies involving distinct populations have confirmed the influence of microbiological aspects of BSIs on the hazards of SS. Consistent with the results of our study, Leibovici et al. conducted a retrospective study involving more than 4000 episodes of bacteraemia in a general population and found that the polymicrobial aetiology was predictive of SS [11]. Moreover, the association of specific BSI by* E. coli* and viridans streptococci with SS in FN patients is feasible because invasive infections by* E. coli* and viridans streptococci are often associated with significant morbidity and mortality [12–15]. For example, the study of Marron et al. [15] reported an association between viridans streptococcal bacteraemia and serious complications, such as SS and acute respiratory distress syndrome, in neutropaenic patients receiving high-dose chemotherapy with cyclophosphamide before allogeneic bone marrow transplantation.

Interestingly, none of the studied clinical characteristics was significantly associated with SS at the time of FN in the multiple logistic regression analysis. These findings differ from the immunocompetent patient studies in which the early development of SS was more frequent in the subjects with advanced age and multiple comorbidities [4–6]. One possible explanation is the relative homogeneity of our study population, which consisted of a large proportion of young patients with haematological malignancies and a relatively low prevalence of associated comorbidities. This fact highlights the need to identify the rapidly available clinical diagnostic features that can predict septic shock in this setting.

This study had some limitations. For example, we found a low incidence of bacteraemia by* Staphylococcus aureus* and* Pseudomonas aeruginosa*, which are often associated with a poorer prognosis in septic patients; therefore, our results should be interpreted with caution, as distinct virulent bacteria may be found in other centres. Furthermore, this study was susceptible to biases that are inherent to observational studies; however, the following factors minimised the possibility of systematic errors: the proper measurement of variables and outcomes with previously defined objective criteria, the use of standardised data collection, the implementation of a followup by a research team that was not related to the care provided, and the use of multivariate analyses.

## 5. Conclusions

The aetiology of BSIs is an important risk factor for SS at the onset of FN in adult cancer patients. Polymicrobial BSI, particularly bacteraemia by* E. coli* and viridans streptococci, are the risk factors for SS at the onset of FN.

Identifying the microbiological factors associated with SS in FN is of paramount importance to clinicians, as this knowledge can determine the preventative measures to avoid BSI by specific highly virulent pathogens and the best choice of empiric antimicrobial therapy.

Future studies are required to assess other possible risk factors for the early onset of SS in the context of FN and to determine whether specific interventions based on the early identification of highly virulent bacteria could result in an effective method to prevent SS and its characteristically pronounced mortality rates.

## Figures and Tables

**Table 1:** Study population characteristics and microorganisms isolated in 115 cases of febrile neutropenia (FN) in hospitalised cancer patients with documented bloodstream infection.

Age, mean years ± SD	42.9 ± 14.1
Female sex	52 (45.2)
Type of cancer	
Acute myeloid leukaemia	59 (51.3)
Acute lymphoblastic leukaemia	19 (16.5)
Chronic myeloid leukaemia	7 (6.1)
Multiple myeloma	11 (9.6)
Lymphoma	15 (13.0)
Other solid tumours	4 (3.5)
Relapsing underlying disease	59 (51.3)
Clinical comorbidity	36 (31.3)
Phase of chemotherapy	
Induction	27 (23.5)
Consolidation	37 (32.2)
Maintenance	26 (22.6)
HSCT	25 (21.7)
Oral mucositis	
Without oral mucositis	59 (51.3)
Grade I	33 (28.7)
Grade II	10 (8.7)
Grade III	6 (5.2)
Grade IV	7 (6.1)
ANC at the time of diagnosis of FN, mean cells/mm³ ± SD	206.1 ± 218.5
Profound neutropenia at the time of diagnosis of FN*	52 (45.2)
Nosocomial-acquired episodes of FN	102 (88.7)
Bloodstream isolates^†^	
*Escherichia coli *	48 (41.7)
Coagulase-negative staphylococci	36 (31.3)
*Klebsiella pneumonia *	13 (11.3)
*Pseudomonas aeruginosa *	11 (9.5)
Viridans streptococci	8 (6.9)
*Enterococcus *spp.	4 (3.4)
*Serratia *spp.	2 (1.7)
*Enterobacter* spp.	2 (1.7)
*Candida *spp.	2 (1.7)
*Salmonella* spp.	1 (0.8)
*Staphylococcus aureus *	1 (0.8)
*Kocuria varians *	1 (0.8)

Data presented as *n* (%) unless otherwise indicated. SD: standard deviation; ANC: absolute neutrophil count; HSCT: hematopoietic stem cell transplantation; *ANC < 100 cells/mm³; ^†^There were 12 cases of polymicrobial bloodstream infections.

**Table 2:** Multidrug-resistant bacteria isolated in 38 cases of bacteraemia in febrile neutropenic patients.

Microorganism	Number isolated (%)
Gram-positive	
MR coagulase-negative staphylococci	25 (65.7)
MR *Staphylococcus aureus *	1 (2.6)
VR *Enterococcus faecalis *	1 (2.6)
Gram-negative	
* Escherichia coli ESBL *	7 (18.4)
* Klebsiella pneumoniae ESBL *	3 (7.8)
*Enterobacter* spp.	1 (2.6)
*Serratia* spp.	1 (2.6)

MR: methicillin resistant; VR: vancomycin resistant; ESBL: extended-spectrum beta-lactamase. There was 1 case of polymicrobial multidrug-resistant bacteraemia.

**Table 3:** Univariate analysis of the risk factors for septic shock (SS) at the time of febrile neutropenia (FN) in hospitalised cancer patients.

Variable	SS group (*n* = 17)	Non-SS group (*n* = 98)	OR (95% CI)	*P* value
Age, years, mean ± SD	43.4 ± 16.0	42.8 ± 13.8	1.00 (0.96–1.04)	0.87
Female sex	10 (58.8)	42 (42.8)	1.90 (0.66–5.41)	0.22
Haematological neoplasm	13 (76.4)	83 (84.6)	0.58 (0.16–2.04)	0.40
Relapsing underlying disease	10 (58.8)	49 (50.0)	1.42 (0.50–4.05)	0.50
Clinical comorbidity	2 (11.7)	34 (34.6)	0.25 (0.05–1.16)	0.07
High-dose chemotherapy regimens*	5 (29.4)	47 (47.9)	0.45 (0.14–1.38)	0.16
Oral mucositis				
Grade I	4 (23.5)	29 (29.5)	0.76 (0.21–2.71)	0.68
Grade II	2 (11.7)	8 (8.1)	1.38 (0.25–7.63)	0.70
Grade III	1 (5.8)	5 (5.1)	1.11 (0.11–10.66)	0.92
Grade IV	1 (5.8)	6 (6.1)	0.92 (0.09–8.63)	0.94
ANC at the time of diagnosis of FN, mean ± SD	161.7 ± 219.0	213.8 ± 218.7	0.99 (0.99–1.00)	0.36
Profound neutropenia at the time of diagnosis of FN^†^	9 (52.9)	43 (43.8)	1.43 (0.51–4.04)	0.49
BSI involving Gram-positive bacteria	6 (39.2)	40 (40.8)	0.79 (0.27–2.31)	0.66
BSI involving Gram-negative bacteria	14 (82.3)	60 (61.2)	2.96 (0.79–10.97)	0.10
Polymicrobial BSI	5 (29.4)	7 (7.1)	5.41 (1.48–19.79)	0.01
MDR BSI	4 (23.5)	34 (34.6)	0.57 (0.17–1.91)	0.37
BSI involving Gram-positive MDR bacteria	3 (17.6)	24 (24.4)	0.66 (0.17–2.49)	0.54
BSI involving Gram-negative MDR bacteria	1 (5.8)	11 (11.2)	0.49 (0.05–4.09)	0.51
BSI by *Escherichia coli *	11 (64.7)	37 (37.7)	3.02 (1.03–8.85)	0.04
BSI by coagulase-negative staphylococci	4 (23.5)	32 (32.6)	0.63 (0.19–2.10)	0.45
BSI by *Klebsiella pneumoniae *	3 (17.6)	10 (10.2)	1.88 (0.46–7.70)	0.37
BSI by *Pseudomonas aeruginosa *	2 (11.7)	9 (9.1)	1.31 (0.25–6.70)	0.73
BSI by viridans streptococci	3 (17.6)	5 (5.1)	3.98 (0.85–18.54)	0.07

Data presented as *n* (%) unless otherwise indicated. ANC: Absolute neutrophil count; BSI: bloodstream infection; HSCT: haematopoietic stem cell transplantation; MDR: multidrug-resistant; OR: odds ratio; 95% CI: 95% confidence interval; SD: standard deviation. *Induction chemotherapy or HSCT; ^†^ANC < 100 cells/mm³.

**Table 4:** Multiple logistic regression analysis of the risk factors for septic shock (SS) at the time of febrile neutropenia (FN) in hospitalised adult cancer patients.

Risk factor	Adjusted OR	95% CI	*P* value
Model 1			
Polymicrobial BSI	5.41	1.48–19.79	0.01

Model 2			
BSI by *Escherichia coli *	4.30	1.27–14.48	0.01
BSI by viridans streptococci	7.58	1.34–42.80	0.02

OR: odds ratio; 95% CI: 95% confidence interval; BSI: bloodstream infection.
